# Genes Related to Sperm Motility Are Under Recent Ongoing Selection in Two Shorebird Species With a Polygynous Mating System and Frequent Multiple Paternity

**DOI:** 10.1111/mec.70493

**Published:** 2026-08-05

**Authors:** Jakob C. Mueller, Bart Kempenaers

**Affiliations:** ^1^ Department of Ornithology Max Planck Institute for Biological Intelligence Seewiesen Germany

**Keywords:** axoneme, *Calidris melanotos*, *Calidris pugnax*, dN/dS test, pectoral sandpiper, rapid evolution, ruff, selective sweep, sperm competition, sperm motility

## Abstract

The analysis of the genomic architecture of selection can be insightful for the understanding of sexual conflict and sexual selection acting in wild populations. In general, there will be a wide range of possible targets of selection in the reproductive system of sexually promiscuous species. In the context of a dynamic conflict, selection is expected to be predominantly recurrent and it might be best investigated by combining population‐level and phylogenetic approaches on multiple species. First, we used population‐based methods to scan for recent selection in the socially polygynous pectoral sandpiper ( *Calidris melanotos* ). We assembled and annotated the pectoral sandpiper genome and re‐sequenced the genome of 20 random individuals of a single breeding population. We identified genes coding for axonemal constituents which are mostly expressed in the testis as selection targets. These genes most likely influence energy‐driven sperm motility, suggesting a role in sperm competition in this species. Second, we replicated the analysis and found similar results in another promiscuous shorebird, the ruff (*Calidris pugnax*), using publicly available data of 25 re‐sequenced individuals. Finally, a comparative genomics approach among six *Calidris* species with publicly available genome assemblies showed similar signals of selection, suggesting historical episodes of postcopulatory sexual selection or sexual conflict in genes related to sperm motility.

## 1 Introduction

Studying historical and contemporary selection acting on genomes in wild populations can provide insight into the evolutionary trajectories of these populations and species. Comparative between‐species studies can detect strong single or potentially long episodes of recurrent positive selection within the phylogenetic history of the analysed species, whereas population‐based approaches are designed to detect recent or ongoing positive selection (Gao 2024). Just by chance, the latter approach applied at a single time point (sampling event) will more often detect long episodes of potentially recurrent selection than unique single selection events in evolutionary history. A combination of population‐based and phylogenetic approaches is therefore ideal to test for recurrent selection in the recent and long‐term history.

Recurrent ongoing selection has been described in dynamic conflict situations such as co‐evolutionary arms races in host–parasite systems or intra‐ and intersexual conflicts. For example, coevolution between hosts and their parasites is expected to follow an arms race leading to recurrent allele fixations in their genomes (Tellier et al. 2014; Cooper et al. 2019). Similarly, postcopulatory sexual selection and sexual conflict can lead to rapidly and repeatedly evolving genes involved in reproduction (Swanson and Vacquier 2002; Clark et al. 2009; Wong 2011). For example, in mammals and birds the observed rapid evolution in the testis is likely explained by positive selection on genes associated with sperm competition (Ramm et al. 2014; Birkhead and Montgomerie 2020; Murat et al. 2023). Sperm competition can result in rapid evolution of reproductive traits related to the female and male reproductive fluid, but also to the assembly, morphology, motility and longevity of the sperm itself (Simmons and Wedell 2020). Multiple studies in birds have investigated the interplay between sperm competition and testis size, sperm morphology and motility (Birkhead and Montgomerie 2020). A meta‐analysis on data stemming mostly from birds and mammals revealed that selection under sperm competition favours longer sperm heads, midpieces and flagella (Lüpold et al. 2020). Given that the flagellum is the component propelling the sperm forward and the midpiece the part generating the necessary energy, this is consistent with the predicted selection for faster sperm (Humphries et al. 2008). However, the molecular underpinnings of the selection associated with sperm competition in birds are understudied. It has been shown that a sex‐linked supergene controls sperm length and velocity in a songbird (Kim et al. 2017). However, studies from mammals and *Drosophila* suggest high variability in the targets of selection between lineages (Wong 2011; Simmons and Wedell 2020). Our study adds information about single genes and their potential functions related to sperm competition in another bird lineage.

Our general aim was to use a genomic approach to investigate selection in a bird species known to be under strong sexual selection. Specifically, we first investigated signatures of recent selection in a population of the pectoral sandpiper ( *Calidris melanotos* ) to assess whether there is detectable rapid evolution in genes related to intense competition for matings and fertilizations. Strong sexual selection through intense male–male competition is indicated by the pronounced sexual size dimorphism in this species, with males being much larger than females (Lesku et al. 2012). Strong male–male competition is also associated with around‐the‐clock activity, whereby more active males sire more offspring (Lesku et al. 2012), and intense breeding site sampling across a large geographical scale (Kempenaers and Valcu 2017). Females of the pectoral sandpiper often mate with multiple males, resulting in a considerable proportion of clutches fertilized by multiple males (27.0% of *N* = 230 complete clutches, our unpublished data). Compared to other socially monogamous shorebirds, pectoral sandpipers also have relatively long sperm (Johnson and Briskie 1999), a trait that relates positively to the intensity of sperm competition (Lüpold et al. 2020).

After finding strong signals of selection in specific gene groups of the pectoral sandpiper while controlling for multiple testing, we replicated our study using publicly available population data on a related species, the ruff (*Calidris pugnax*). This species has a similar mating system, including intense male–male competition (Lank et al. 2002) and extensive breeding site sampling (Kempenaers et al. 2025). Moreover, females are often promiscuous, leading to frequent multiple paternity (51.5% of *N* = 66 complete clutches, Thuman and Griffith 2005). Ruffs also have exceptionally long sperm (Johnson and Briskie 1999; Bulla et al. 2024). We specifically tested whether there is evidence for ongoing selection in the same gene groups found to be under selection in the pectoral sandpiper.

Third, we used a phylogenetic approach to investigate whether historical episodes of selection are consistent with the detected recent selection in the context of sexual selection and conflict in the pectoral sandpiper and the ruff. We conducted a comparative genomic study based on six species including four additional *Calidris* species with publicly available reference genome assemblies and with contrasting mating systems. The spoon‐billed sandpiper ( *C. pygmaea* ) and the dunlin ( *C. alpina* ) are considered socially monogamous, whereby sperm competition supposedly plays a minor role. The Temminck's stint ( *C. temminckii* ) and the little stint ( *C. minuta* ) are described as having a variable social mating system including monogamy, and successive polygyny and polyandry (van Gils et al. 2020a, 2020b).

## 2 Methods

### 2.1 Reference Genome Assemblies and Gene Annotation

We assembled a 1.17 Gb long pectoral sandpiper reference genome using linked‐read data (10× Genomics) sequenced at the National Genomics Infrastructure (NGI), Sweden, with a read coverage of 57×. The reference comes from a male, which we blood sampled in 2018 at our study site near Utqiagvik (Barrow, 71° 18′ N, 156° 44′ W), at the northern tip of the Alaskan Arctic Coastal Plain (for details of the study site and the general procedures see Lesku et al. 2012 and Kempenaers and Valcu 2017). We stored the blood in ethanol and extracted the DNA with phenol/chloroform. The assembly produced by the Supernova software (Weisenfeld et al. 2017) consisted of 20,548 scaffolds with an N50 of 10.1 Mb and with 92.0% assigned BUSCO complete single‐copy genes (of aves_odp12 database). The assembly is deposited under NCBI accession number BioProject PRJNA1379596.

The ruff reference genome assembly (GCA_036321245.1) is 1.1 Gb and has been published by Hill et al. (2023) under the NCBI BioProject accession number PRJNA816664. It is based on a satellite male, sampled in Sweden in 2016, sequenced by a long‐read technique (PacBio). The 463 scaffolds have an N50 of 26.3 Mb, and 94.3% of BUSCO complete single‐copy genes have been assigned.

For the phylogenetic analysis, we downloaded assemblies from the NCBI genome database for the spoon‐billed sandpiper (ASM369795v1), the dunlin (ASM3362846v1), the Temminck's stint (ASM3362836v1), and the little stint (ASM3362842v1).

To make gene annotation consistent between species, we started with the well‐annotated ruff genome (ASM143184v1, NCBI Annotation Release 100, Lamichhaney et al. 2016). The 16,523 protein‐coding genes of the ruff have been annotated by the NCBI Eukaryotic Genome Annotation Pipeline including transcript and protein alignments with all major bird models, *Xenopus* and humans, and with novel gene model predictions. We transferred these gene models to the reference genome assemblies of all six *Calidris* species described above using GeMoMa (Keilwagen et al. 2019). This gene model mapper utilizes the homology of the coding sequences and the conserved intron positions among related species to transfer homologous gene models and make them comparable among the studied species. With this method, we annotated 10,495 protein‐coding genes in our pectoral sandpiper genome assembly (File S1), 12,326 genes in the newly published ruff assembly, 13,012 genes in the spoon‐billed sandpiper assembly, 10,241 genes in the dunlin assembly, 10,381 genes in the Temminck's stint assembly, and 9098 genes in the little stint assembly.

### 2.2 Population Samples and Resequencing Data

Between 2005 and 2009, we caught male pectoral sandpipers at their breeding site between late May and late June using a mistnet held by two people. From each individual, we took a 200–300 μL blood sample from the brachial vein and stored it in Queen's lysis buffer. From these samples, we randomly selected 20 for sequencing. We extracted genomic DNA from the blood with the NucleoSpin Blood Quick Pure kit (Macherey‐Nagel) and sent the samples to the Sequencing Core Facility of the Max Planck Institute for Molecular Genetics in Berlin, where all samples were sequenced in a paired‐end library with 500 bp fragment size and a short‐read length of 125 bp (Illumina HiSeq). The final read coverage per sample was about 21×.

For the ruff, we utilized the published resequencing data of 25 males of the three genetically determined morphs (15 independents, 9 satellites and one faeder; Lamichhaney et al. 2016). The birds were caught on leks (male display sites) on the island of Gotland (Sweden) in the Baltic Sea (57° 10′ N, 18° 20′ E) using cannon netting during the breeding seasons of 1990–2002. The sequencing library was also based on 500 bp fragments and sequenced to 2 × 125‐bp paired‐end reads with a coverage of about 8×. The reads were downloaded from the NCBI accession SRA266458.

### 2.3 SNP Calling

First, we mapped the sequence reads of each individual to the reference genome of the same species using minimap2 (Li 2018). We then deduplicated the reads with sambamba (Tarasov et al. 2015) and called the SNPs in the populations using freebayes (Garrison and Marth 2012) with stringent input and mapping quality filters. SNPs were filtered for quality (phred‐like score > 20), no “missings” (all individuals with called genotypes), and overall read depth within the 5% and 95% quantiles. We identified 34,382,334 biallelic SNPs for the pectoral sandpiper and 3,783,958 biallelic SNPs for the ruff. The difference in the number of SNPs is due to the different read depths between the two species.

### 2.4 Demographic Inference

Because selection signatures can be compromised by genetic drift effects (Cheng and Steinrücken 2024), we adjusted our selection scans to population‐specific demographic models, which we estimated from genome‐wide linkage data and site frequency spectra. We inferred the recent demographic history of the pectoral sandpiper and ruff populations utilizing linkage disequilibrium (LD) information at different genetic distances as implemented in the software GONE (Santiago et al. 2020). The fundamental idea is that LD between distant loci depends more strongly on recent genetic drift, whereas LD among nearby loci can be more informative about genetic drift in distant times. In general, however, the magnitude of LD at any given genetic distance is the result of the cumulative effects of genetic drift over all previous generations (Santiago et al. 2020). The software repeats a genetic optimization algorithm in 40 independent runs to search for a global optimal solution of the historical population size series that best fits the observed LD values. We selected large scaffolds (> 15 Mb) for the estimation of recent historical effective population size (N_e_), because theory predicts that LD between markers of more than 10 Mb distance on chromosomes with a recombination rate of 2 × 10^−8^ is already affected by the population size 2.5 generations ago (Hayes et al. 2003; Boitard et al. 2016; Santiago et al. 2020).

We also estimated recent demographic history of the pectoral sandpiper and the ruff with a different approach utilizing site frequency spectrum (SFS) information of the large scaffolds (> 15 Mb) as implemented in the software fastsimcoal2 (Excoffier et al. 2013). We ran 100,000 simulations and applied parametric bootstrapping to obtain 95% confidence intervals.

### 2.5 Selection Scans

We scanned the genome of the pectoral sandpiper and the ruff for selective sweeps in consecutive 20 kb windows using diploS/HIC in the diploid mode (Kern and Schrider 2018). The standard model distinguishes between neutral scenarios and two types of sweeps: hard sweeps originating from *de novo* beneficial mutations and soft sweeps originating from standing variation which becomes beneficial (Messer and Petrov 2013). Afterwards, we pooled the hard and soft sweeps as suggested for situations where evidence for selection is weak to moderate (Kern and Schrider 2018). To characterize sweep evolution, the supervised machine‐learning algorithm employs twelve major summary statistics (normalized values) of selective sweeps including diversity measures, allele frequency spectra, linkage‐disequilibria patterns, and haplotype structures in the focal and ten neighbouring 20 kb windows (Kern and Schrider 2018).

For the learning step we produced 46,000 genomic simulations of 220 kb (eleven 20 kb windows) for the neutral (including neighbouring windows to sweeps), hard sweep and soft sweep scenarios. For these coalescent simulations we used discoal (Kern and Schrider 2016), assuming a constant effective population size N_e_ of 200,000 over time for the two species (see results about demographic history and Figures S1 and S2), a selection coefficient (s) between 0.001 and 0.01 and—in the case of soft sweeps—a starting minor allele frequency of 0.05. Such moderate selection coefficients are within the estimated range in studies of humans and are strong enough to be effective (i.e., N_e_s > 1) in populations with N_e_ > 10,000 (Kern and Schrider 2018; Eyre‐Walker and Keightley 2007). We then extracted 2000 feature vectors of the twelve summary statistics in each window for each of the neutral and sweep scenarios and trained the model. After this learning step, we used the model to predict the selective sweep state of 20‐kb windows in the scaffolds larger than 220 kb, based on their empirical feature vectors. We used the R package CMplot (Yin et al. 2021; R Core Team 2020) to produce a Manhattan plot of sweep probabilities based on the model predictions.

### 2.6 Comparative Genomics

Using the set of six available *Calidris* genomes we tested for all shared genes whether they have experienced positive selection at least at one site on one phylogenetic branch. We used a likelihood ratio test to compare a model which allows for positive selection at some sites (i.e., ratio of non‐synonymous to synonymous substitutions dN/dS > 1) against a constrained model without potential positive selection, as implemented in the software BUSTED (Murrell et al. 2015). This “stochastic selection” test has sufficient statistical power to detect episodic or localized selective events, even for relatively short genes and small sample sizes (Murrell et al. 2015). Although not generally recommended (Wisotsky et al. 2020), we applied the parsimonious model without synonymous substitution rate variation (SRV), because it was preferred over the model with SRV in 67% of the tests (based on small‐sample AIC_c_ values). This outcome might be due to the small tree length (and therefore low observed substitution counts per site) in our dataset in comparison to the examples in Wisotsky et al. (2020). Before testing, we codon‐aligned the DNA sequences of all genes using Prank (Löytynoja 2014). An example guide tree is shown in Figure S3.

### 2.7 Functional Enrichment Analysis

We tested for enrichment of associated gene functions of high selective sweep probabilities across the genome using the gene ontology (GO) annotation. We used the selected list of 3348 GO‐slim terms defined by PANTHER (Mi et al. 2019) as being both informative of function and evolutionarily conserved. Windows of the selection scans were associated to GO terms via overlapping genes or when lying within a maximum distance of 5 kb to genes (whole regions including exons and introns). If a gene (as defined by a gene symbol) covered more than one window, we calculated a mean sweep probability. We used the Wilcoxon rank‐sum test implemented on the Panther website (https://www.pantherdb.org/) to test for GO term enrichment in genes with relatively high mean selective sweep probabilities in comparison to all tested genes. We accounted for multiple tests across the many GO terms by calculating the false discovery rate (FDR) according to the Benjamini‐Hochberg procedure. Gene‐concept networks were produced with the R package enrichplot (Wu et al. 2021).

In the comparative genomic analysis, we tested functional enrichment of the genes under stronger selection using the Wilcoxon rank‐sum test on the inverted *p*‐values of all selection tests (BUSTED). Due to the high number of rank ties (equally ranked genes with little substitutions), we employed a permutation procedure to calculate a family‐wise error rate (FWER) as implemented in the R package GOfuncR (Grote 2018) to correct for testing multiple GO terms.

## 3 Results

### 3.1 Demographic Inference

For the pectoral sandpiper, the estimated local effective population size (N_e_) based on LD measures was 5,000,000 (the default upper limit of the program) for the last 400 generations (Figure S1A). In general, however, there is no predictive power to precisely estimate N_e_ larger than about 100,000 based on LD measures with small samples, especially for recent generations (E. Santiago, personal communication). The SFS‐based method estimates recent effective population sizes of the pectoral sandpiper in the range of 150,000 to 400,000 (Figure S2A).

Similarly, the estimated local effective population size for the ruff was 5,000,000 using the LD‐based method and between 200,000 and 400,000 using the SFS‐based method for the last several hundred generations (Figures S1B and S2B).

Accurate census estimates of total population size have proven difficult to obtain for shorebird species, which display high temporal and spatial variability in population density, disperse widely during migration, and are distributed over large, inaccessible areas during the breeding and non‐breeding seasons (Farmer et al. 2020). Rough census estimates of the entire pectoral sandpiper population range from 500,000 to 1,600,000 (Brown et al. 2007). The total population size of the ruff was estimated based on census data to be as high as 2,000,000 (van Gils, Wiersma, and Kirwan 2020b).

We have thus set local effective population size for the simulations for the selection scans to a constant N_e_ of 200,000 in both species. This effective population size is still manageable in the simulations, while also being large enough to have negligible influence on the selection scans.

### 3.2 Population‐Level Selection Signatures

**FIGURE 1:**
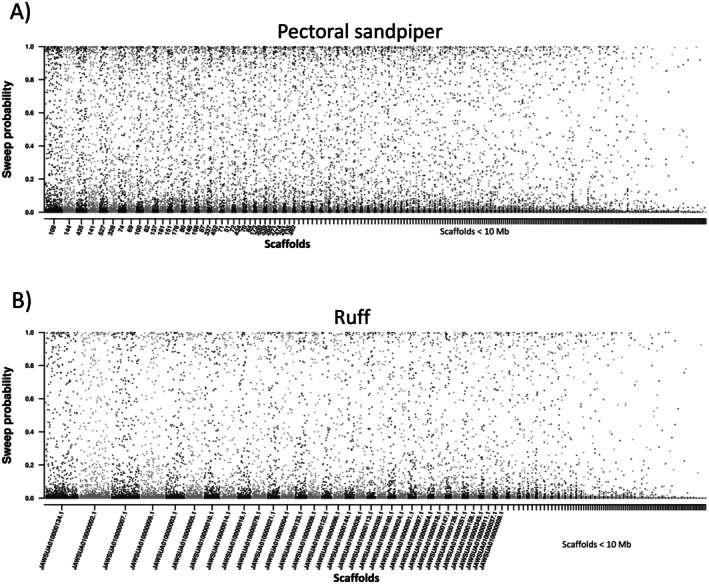
Manhattan plot showing for each tested genomic window the selective sweep probability for the pectoral sandpiper (A) and the ruff (B).

**FIGURE 2:**
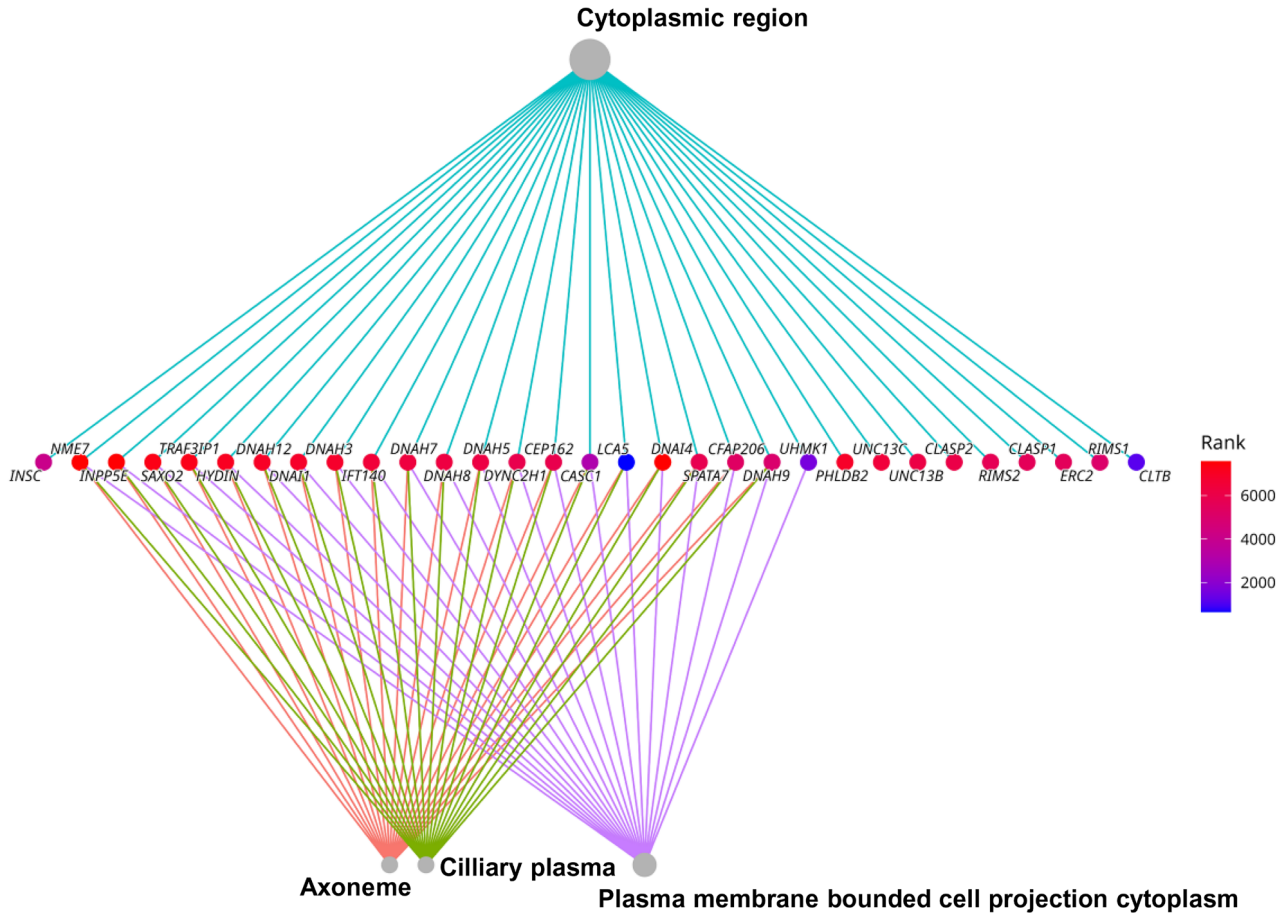
Gene‐concept network showing the significant GO terms of the functional enrichment test for genes with evidence for strong selection in a pectoral sandpiper population. Dot colour reflects the ranking of the annotated genes by mean selection probability (red refers to high ranking and blue to low ranking).

In the pectoral sandpiper, we evaluated selection sweep probabilities for 49,456 20‐kb windows in 246 scaffolds of sufficient size and nucleotide variant density. Windows with high sweep probabilities were candidates for strong sweeps, whereas the remaining windows were predicted to be neutral or neighbouring to sweeps, or the evidence was inconclusive (low probabilities for all scenarios). The windows with evidence for strong sweeps were randomly distributed across the genome (Figure 1A; all 7814 tested genes and their mean, minimum and maximum sweep probabilities for the overlapping 20 kb windows are listed in File S2). When ranking genes by mean sweep probability, the higher‐ranking genes were significantly enriched for functioning in the “axoneme” or “ciliary plasm” (Figure 2). The genes annotated to these GO terms are also part of the parent terms “plasma membrane bounded cell projection cytoplasm” and “cytoplasmic region”. These terms also show up as significant in the enrichment tests (Table S1).

In the ruff, we calculated sweep probabilities for 51,435 20‐kb windows in 125 scaffolds. Similar to the pectoral sandpiper, the windows showing evidence for strong sweeps appear to be randomly distributed across the genome (Figure 1B; all 10,760 tested genes and their mean, minimum and maximum sweep probabilities for the overlapping 20 kb windows are listed in File S3). The higher‐ranked genes (based on mean sweep probability) were not significantly enriched for any GO term after table‐wise correction for multiple testing (Table 1). However, the same four GO terms found to be significant for the pectoral sandpiper were associated with the four lowest and highly significant single‐term *p*‐values among the 3348 tested terms (Table 1). Thus, the results from the ruff can be considered a positive replication of the results from the pectoral sandpiper indicating axoneme genes as selective targets. The weaker signal in the ruff might be due to the lower read coverage and thus lower number of called SNPs in comparison to the pectoral sandpiper.

**TABLE 1:** Functional enrichment in selective sweep regions of the ruff according to the Wilcoxon rank‐sum test of mean sweep probabilities per gene. Panther GO‐Slim annotation with parent terms indented. All terms with FDR < 0.5 are listed. The four lowest raw *p*‐values (in bold) are within the “Axoneme” group. Genes with mean sweep probability in upper 20th percentile shared with pectoral sandpiper are in bold.

GO‐term	FDR	Raw *p*‐value	Annotated genes	Genes with mean sweep prob. in upper 20^th^ percentile
Axonemal dynein complex (CC)	0.34	0.0049	8	***DNAH12**, **DNAI1**, **DNAH3***
Axoneme (CC)	0.38	**0.00079**	31	*CFAP206, CCDC113, **SAXO2**, EFHC1, **DNAH12**, CEP162, **DNAI1, DNAH3, IFT140***
Ciliary plasm (CC)	0.28	**0.0011**	32	*CFAP206, CCDC113, **SAXO2**, EFHC1, **DNAH12**, CEP162, **DNAI1, DNAH3, IFT140***
Plasma membrane bounded cell projection cytoplasm (CC)	0.36	**0.0022**	33	*CFAP206, CCDC113, **SAXO2**, EFHC1, **DNAH12**, CEP162, **DNAI1, DNAH3, IFT140***
Cytoplasmic region (CC)	0.28	**0.0023**	47	*CFAP206, CCDC113, **SAXO2**, EFHC1, **DNAH12**, CEP162, CLTA, **DNAI1, DNAH3**, RIMS2, CLASP1, **IFT140***
Microtubule cytoskeleton (CC)	0.37	0.0062	252	*DNAH14,CFAP206,IFT88,CCDC113,DYNC1LI1,TBC1D31,MAP10,KIF27,**SAXO2**,CCNJL,TUBGCP5,HAUS3,EFHC1,UNC119,CNTRL,CEP164,SKA2,DYNLT3,CLUAP1,EML4, SEPT9,DNM1,KATNB1,**DNAH12**,RMDN3,DNM1L,BRSK2,CCDC88C,SDCCAG8, AHI1,GAS8,ARL3,CEP162,SEPT7,**DNAI1**,HAUS8,CCDC88A,DZIP1L,CENPF,TTC26, **DNAH3**,RMDN2,CEP128,KIF6,PPP2R3C,CEP126,KIF15,KIAA0586*,*KIF4A,KIF16B, CLASP1,KIF14,CEP120,TCTEX1D1,CLIP2,CEP97,CKAP2,EML1,KATNAL1,CEP170, MPHOSPH9,SPAG6,POC1A,CDC14A,**IFT140**,CCNB1*
Cortical actin cytoskeleton (CC)	0.30	0.0032	11	*RTKN2, SHROOM3, HIP1R, SHROOM2, LLGL2, ACTN1*
Cell cortex (CC)	0.49	0.012	60	*GPSM2,RTKN2,EXOC7,SHROOM3,HIP1R,SEPT9,SHROOM2,FRYL,CLTA,SEPT7,LLGL2,RIMS2,CLASP1,ACTN1,CLIP2,CAPZA2,EXOC6*
Cul4‐RING E3 ubiquitin ligase complex (CC)	0.28	0.0035	11	*DCAF17, CUL4A, CRBN, DCAF5, VPRBP, DCAF6*
Cullin‐RING ubiquitin ligase complex (CC)	0.46	0.013	56	*ANAPC16,FBXO15,DCAF17,CUL4A,CRBN,CUL3,DCAF5,VPRBP,DCAF6,ANAPC10, FBXO25,KCTD17,FBXL17*
Perikaryon (CC)	0.42	0.0088	3	*GHRH, TH, SLC5A7*
High‐density lipoprotein particle (CC)	0.46	0.014	4	*APOA1, APOA4, APOA5*

We identified 5 genes that are part of the significant GO terms with mean sweep probabilities in the upper 20th percentile in both the pectoral sandpiper and the ruff: *DNAH3, DNAH12, DNAI1, SAXO2, and IFT140*. These genes have one 20‐kb window classified as a sweep with high probability (0.45–0.99) in both species, except for one (*IFT140*) which is classified as linked to a sweep in the ruff (Files S2 and S3). In 4 of these 5 loci, the selective sweep was localized in the same gene region in both species (Figure S4). For each of the 5 genes in the pectoral sandpiper and the ruff, Figure S5 shows heatmaps of the twelve summary statistics (as normalized values) that were used for the selection scans for the selective sweep window and for ten neighbouring 20‐kb windows. The majority of the 5 genes show the highest level of expression (among the tested tissues) in the testis in adult male ruffs and in humans (Table S3). Note that the 5 genes are located on 5 different scaffolds in both the pectoral sandpiper and the ruff, indicating independent selection events (Table S3).

### 3.3 Phylogenetic Selection Signatures

**FIGURE 3:**
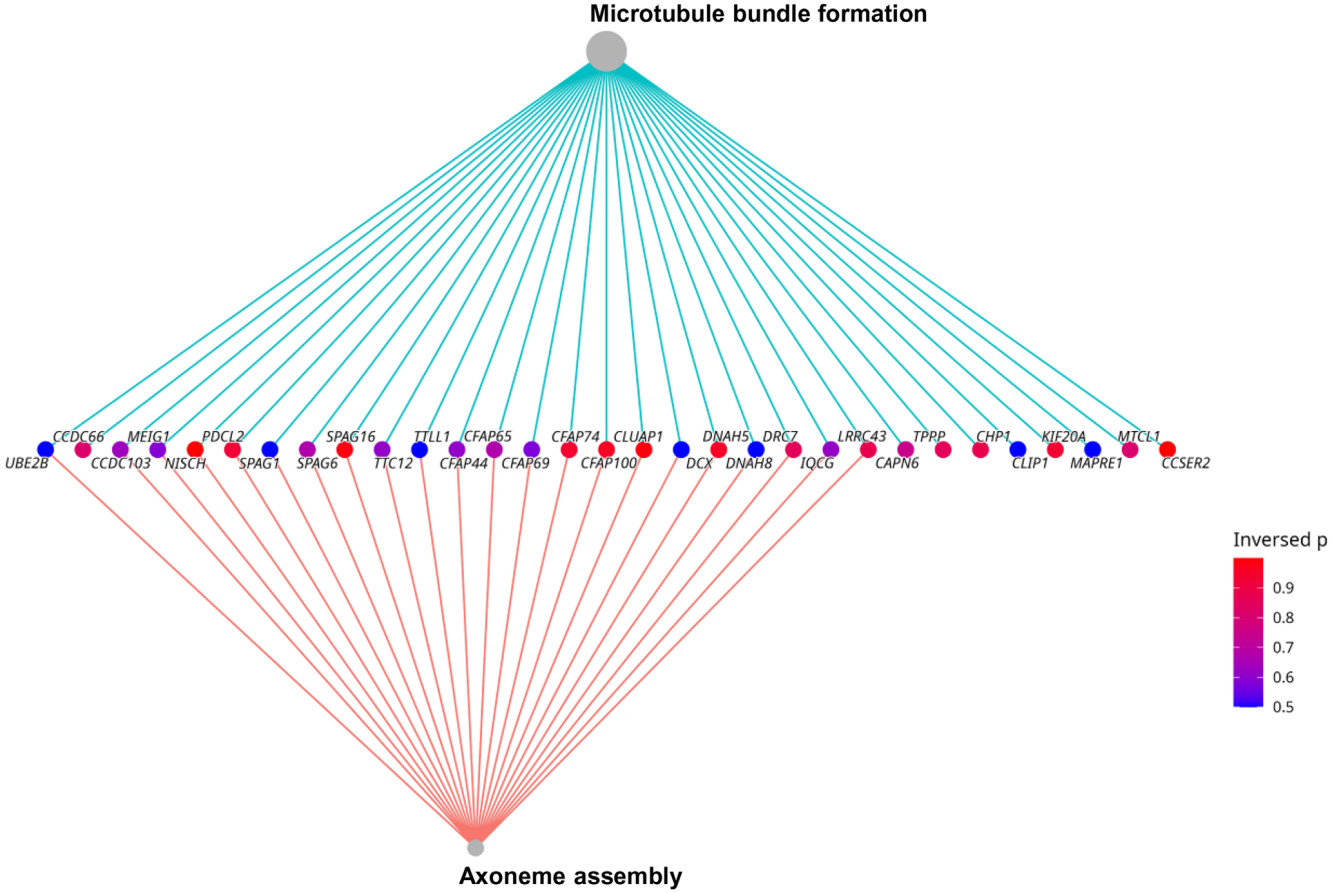
Gene‐concept network showing the significant GO terms of the functional enrichment test for genes with evidence for strong selection in the phylogenetic tree based on six *Calidris* species. Dot colour reflects the inversed *p*‐value of the phylogenetic test for each annotated gene (red indicates a high inversed *p*‐value and blue a low inversed *p*‐value).

We tested 5130 shared genes for signals of selection in the phylogenetic setting of the six *Calidris* species with available genome assemblies (see File S4 for all single gene results). A rank test on the inverted *p*‐values revealed two significantly enriched GO terms associated with higher ranked genes (Figure 3). The two terms are “axoneme assembly” and “microtubule bundle formation” (Table S2).

## 4 Discussion

Using genomic data from a population of pectoral sandpipers, we identified genes related to the assembly and functioning of the axoneme as selection targets. We then replicated this finding by analysing published genomic data from a ruff population. A phylogenetic genomic study on six *Calidris* species independently provided evidence for selection on axoneme‐related genes. In sum, two tests based on polymorphisms in populations of the pectoral sandpiper and the ruff and one test based on substitutions among six *Calidris* species indicate strong evidence for selection in the axoneme complex.

The axoneme is a microtubule‐based architecture in the internal core of cilia and flagella. Motile cilia function in various tissues such as respiratory tracts, lungs, kidneys, oviducts, brains and sperm and are composed of more than 600 proteins (Ishikawa 2017; Pazour 2024). Most of the genes have also been described in the avian spermatozoa (Wang et al. 2023). The highlighted 5 genes with axoneme function and high selection probabilities in both the pectoral sandpiper and the ruff are characteristic components of the sperm flagellum. In an effort to describe the complete human sperm proteome, Castillo et al. (2018) and Greither et al. (2023) listed about 9300 genes. Four of our 5 highlighted genes are included in this list (Table S3). All 5 genes show high expression in the testis of ruffs (Loveland et al. 2025) and humans (Fagerberg et al. 2014), in most cases being the tissue with the highest expression among the tested tissues (Table S3). Thus, it is highly likely that the 5 highlighted genes, but also the other species‐specific high‐ranked genes of the enriched “axoneme” group, play an important role in the evolution of the sperm flagellum.

Specific functions are involved in cilium assembly (*SAXO2, IFT140*) and cilia force‐production/motility (*DNAH12, DNAH3, DNAI1*) (NCBI gene db). Some of these genes and other members of the dynein axonemal chain (*DNA*) family—dynein motor proteins driven by ATP‐induced conformational changes (Ishikawa 2017) – are reported to be involved in male infertility (Inaba 2011; Zhou et al. 2024; Geng et al. 2024). Direct genotype–phenotype association studies are needed to identify the variants under selection and their molecular underpinning (non‐synonymous, splicing or regulatory sites). The regional co‐localization of selective sweeps within the compared genes in pectoral sandpipers and ruffs (Figure S4) indicate that specific protein domains are the targets of selection. These may include hydrolytic ATP binding sites among potentially many other functional sites (Geng et al. 2024).

Axoneme genes functioning in the sperm flagellum are prime candidates for being targeted by sexual selection in the context of sperm competition. Sperm competition can cause continuous postcopulatory selection on males, but it can also lead to sexually antagonistic selection when females moderate or counteract male sperm competition in their reproductive tract (Parker 2020; Birkhead and Montgomerie 2020). It is plausible that such postcopulatory selection processes can produce consistent arms races among the involved genes (Gage 2004; Simmons and Wedell 2020). Due to the multiple potential gene targets and lineage‐specific idiosyncratic selection regimes, Wong (2011) suggested to apply a combination of comparative and population genome‐wide studies to investigate reproductive tract protein evolution. Our study indicates that recurrent selection in the context of sperm competition and sexual conflict can be detected by population genetic scans.

Sperm competition and sexual conflict should be more prominent in species or populations with a promiscuous mating system (Birkhead and Montgomerie 2020). In shorebirds, sperm morphology (especially tail and midpiece length) correlates with the intensity of sperm competition (Johnson and Briskie 1999). Species with polygynous or polyandrous mating systems, including the lekking ruff and the polygynous pectoral sandpiper, had longer sperm tails compared to socially monogamous species (Johnson and Briskie 1999). Our study indicates that promiscuous sandpipers may not only have longer sperm, but also a modified force‐producing machinery in the sperm. Although we find clear signals of selection on genes related to sperm motility, the direction of the identified selection and the underlying phenotypic traits cannot be deduced from our study. In the context of sperm competition and post‐copulatory female choice one can speculate about selection for increased sperm swimming speed (e.g., Fitzpatrick et al. 2009), but also for other traits, such as sperm longevity or morphology, or traits to counter‐act selection on females driven by sexual conflict (e.g., the evolution of sperm hyper‐activation, Cooper and Phadnis 2017). A recent study on several avian brood parasites identified an enrichment of genes under positive selection that are related to sperm development and movement, including dynein axonemal chain (*DNA*) protein genes of the sperm flagellum (Osipova et al. 2026). The authors suggest that their finding can be explained by increased sperm competition in parasitic lineages following the loss of parental care.

In summary, a genome‐wide scan for recent or contemporary signals of selection in two promiscuous sandpiper species as well as historical selection in six species of the *Calidris* genus revealed evidence for selection on genes functioning in the axoneme of sperm flagella. These genes might represent important targets of sexual and/or sexually antagonistic selection in the context of sperm competition in sandpipers and in other species.

## Author Contributions

J.C.M. and B.K. contributed to study conception and design. J.C.M. performed all data analyses. J.C.M. and B.K. drafted the final manuscript.

## Funding

This work was supported by the Max Planck Society (to B.K).

## Disclosure

Benefits from this research accrue from the sharing of our data and results in public databases as described above.

## Conflicts of Interest

We declare we have no competing interests.

## Supporting information


**File S1:** Gene annotation of pectoral sandpiper genome.
**File S2:** diploS/HIC results in pectoral sandpiper.
**File S3:** diploS/HIC results in ruff.
**File S4:** BUSTED results in 6 Calidris species.
**File S5:** Commands and scripts.

**Figure S1:** Estimated effective population size (N_e_) calculated by a LD‐based method (GONE) from the present to 400 generations back in time for the pectoral sandpiper (A) and the ruff (B). Note that all 40 runs during the global optimization step indicate the same single trajectory as shown. The range of estimates for single large scaffolds (> 15 Mb) is shown in grey. The default upper estimation limit is 5,000,000.
**Figure S2:** Estimated effective population size (N_e_) calculated by a SFS‐based method (fastsimcoal2) from the present to 400 generations back in time for the pectoral sandpiper (A) and the ruff (B). 95% confidence intervals based on parametric bootstrapping are shown in grey.
**Figure S3:** Example neighbour‐joining guide tree for the alignment of the *DNAH12* coding sequences of the six *Calidris* species produced by PRANK.
**Figure S4:** Genomic regions indicating the selective sweep window and the detected non‐synonymous SNPs (NS‐SNPs) for the pectoral sandpiper and the ruff in the genes *DNAH12* (A), *DNAI1* (B), *DNAH3* (C), *SAXO2* (D) and *IFT140* (E). Note that closely located NS‐SNPs cannot be discriminated in the plot. The colour in each SNP symbol represents the allele frequency (blue = reference allele, red = alternative allele).
**Figure S5:** Heatmaps of the twelve summary statistics (as normalized values) used for the selection scans in the selective sweep window (win5) and ten neighbouring 20‐kb windows for the pectoral sandpiper and the ruff in the genes *DNAH12* (A), *DNAI1* (B), *DNAH3* (C), *SAXO2* (D) and *IFT140* (E). The summary statistics are *p* (pi), θ_w_ (thetaW), Tajima's D (tajD), genotype distance variance (distVar), genotype distance skew (distSkew), genotype distance kurtosis (distKurt), number of multilocus genotypes (nDiplos), multilocus genotype equivalents of H_1_, H_12_ and H_2_/H_1_ (J_1_, J_12_, J_2_/J_1_, respectively), unphased Z_nS_ (ZnS), and unphased ω (Omega) (see Kern and Schrider 2018).
**Table S1:** Functional enrichment in selective sweep regions of the pectoral sandpiper according to the Wilcoxon rank‐sum test of mean sweep probabilities per gene. Panther GO‐Slim annotation with parent terms indented. All terms with FDR < 0.05 are listed. Genes with mean sweep probability in upper 20th percentile shared with ruff are in bold.
**Table S2:** Functional enrichment in episodic selection across the phylogeny of six *Calidris* spp. according to the Wilcoxon rank‐sum test on inversed *p*‐values (1‐*p*) of the unrestricted branch‐site dN/dS tests (BUSTED) per gene. Panther GO‐Slim annotation with parent terms indented. All terms with FWER < 0.05 are listed.
**Table S3:** Expression of the 5 high‐lighted shared genes in adult male ruff tissues and humans, and scaffold position in pectoral sandpiper and ruff genome. Values represent DESeq2‐normalized read counts (mean ± sd). Note, brain represents an average over 7 brain areas. Ruff data are from Loveland et al. 2025 and human tissue expression data from Fagerberg et al. (2014) on 27 different tissues (NCBI gene db). Human sperm proteome according to Greither et al. 2023 and Castillo et al. 2018.

## Data Availability

The new assembly of the pectoral sandpiper and population sequence data are deposited at the NCBI archive under BioProject number PRJNA1379596. File S1 lists the annotation of all gene models in the pectoral sandpiper. The commands and scripts are compiled in File S5.
