# Supplementary material for: Genes Related to Sperm Motility Are Under Recent Ongoing Selection in Two Shorebird Species With a Polygynous Mating System and Frequent Multiple Paternity
Source: Mol Ecol. 2026 Aug 5;35(15):e70493. doi: 10.1111/mec.70493 (PMC13439353; doi:10.1111/mec.70493)
Supplement: Supplementary file 1 [file MEC-35-e70493-s001.zip › mec70493-sup-0002-Supinfo2/mec70493-sup-0002-FigureS4-S5-TableS1-S3@MEC-9557357_Supplemental_Figures_Tables.pdf]

MOLECULAR ECOLOGY

Supplemental Figures and Tables for:

Genes related to sperm motility are under recent ongoing selection in two shorebird species with a polygynous mating system and frequent multiple paternity

Jakob C. Mueller, Bart Kempenaers

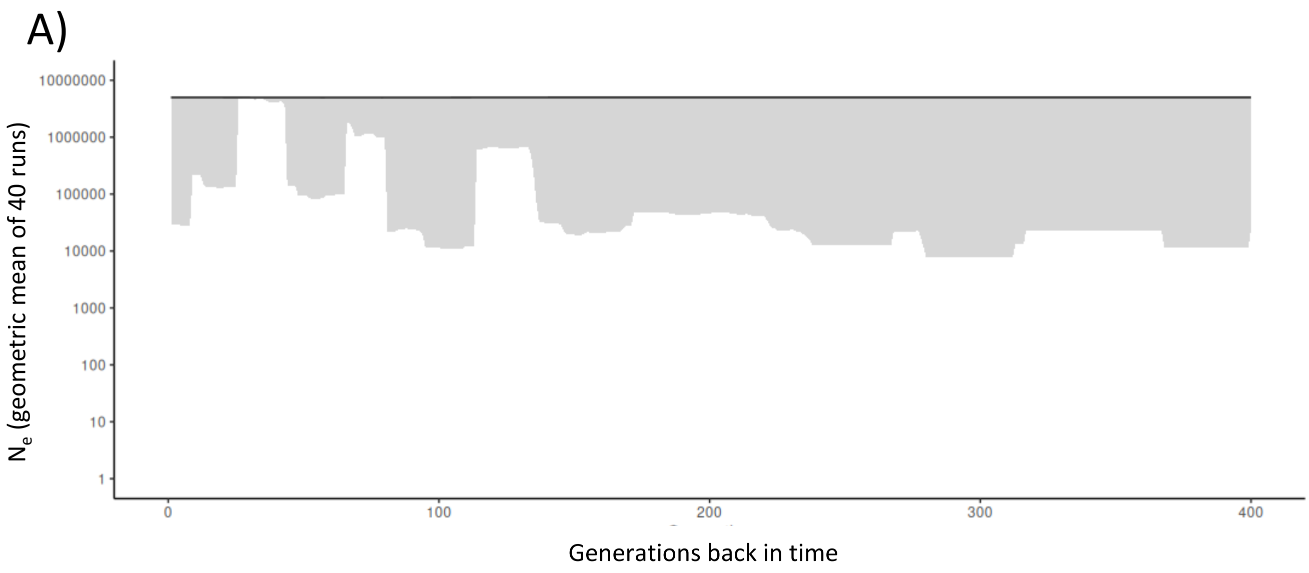

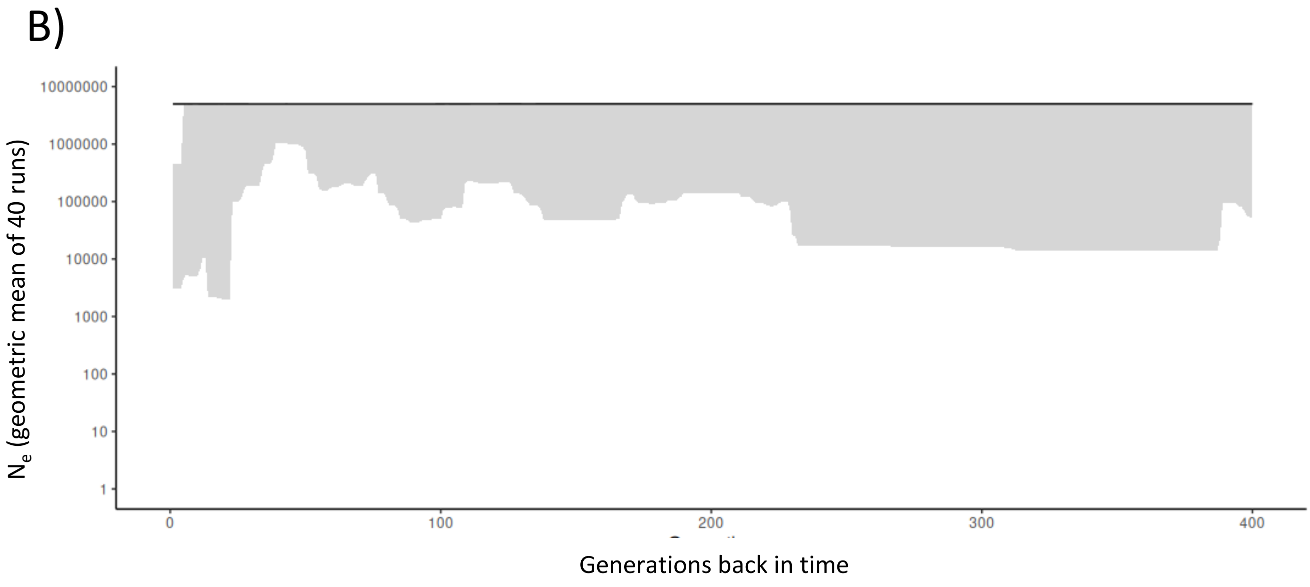

Suppl. figure 1. Estimated effective population size (N_e) calculated by a LD-based method (GONE) from the present to 400 generations back in time for the pectoral sandpiper **(A)** and the ruff **(B)**. Note that all 40 runs during the global optimization step indicate the same single trajectory as shown. The range of estimates for single large scaffolds (> 15 Mb) is shown in grey. The default upper estimation limit is 5,000,000.

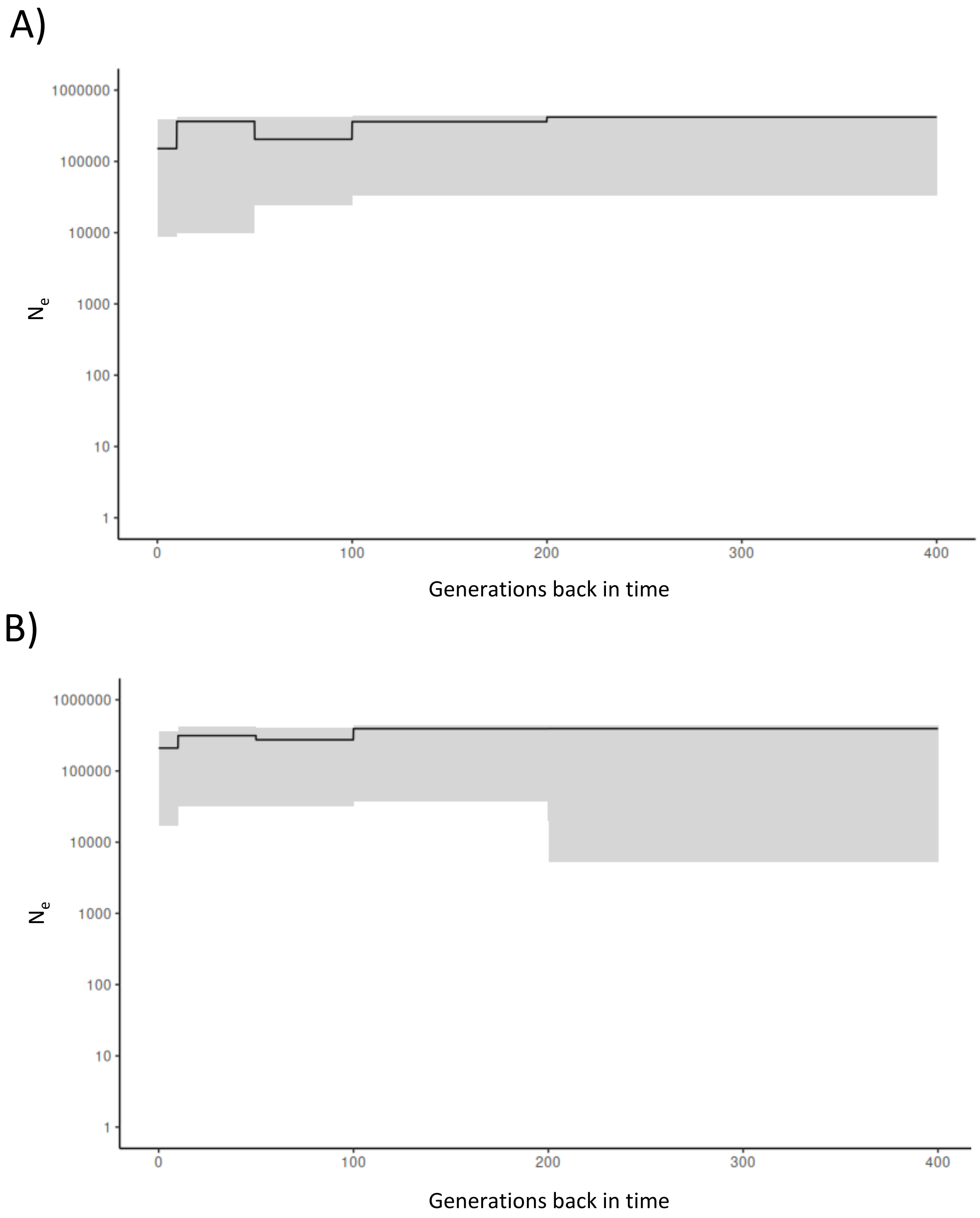

Suppl. figure 2. Estimated effective population size (N_e) calculated by a SFS-based method (fastsimcoal2) from the present to 400 generations back in time for the pectoral sandpiper **(A)** and the ruff **(B)**. 95% confidence intervals based on parametric bootstrapping are shown in grey.

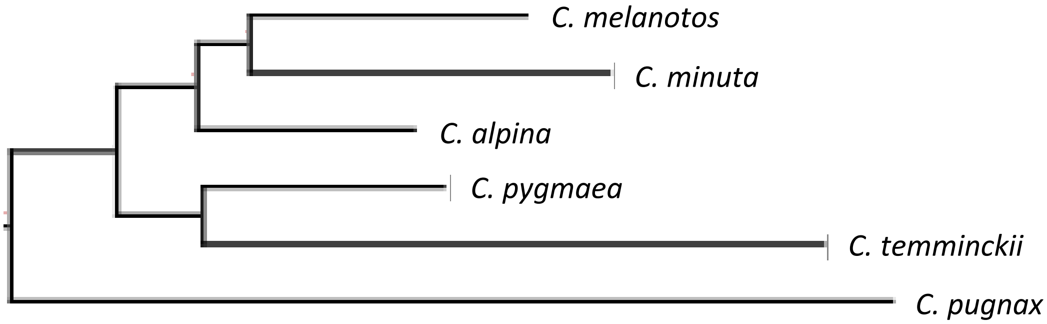

Suppl. figure 3. Example neighbour-joining guide tree for the alignment of the *DNAH12* coding sequences of the six *Calidris* species produced by PRANK.

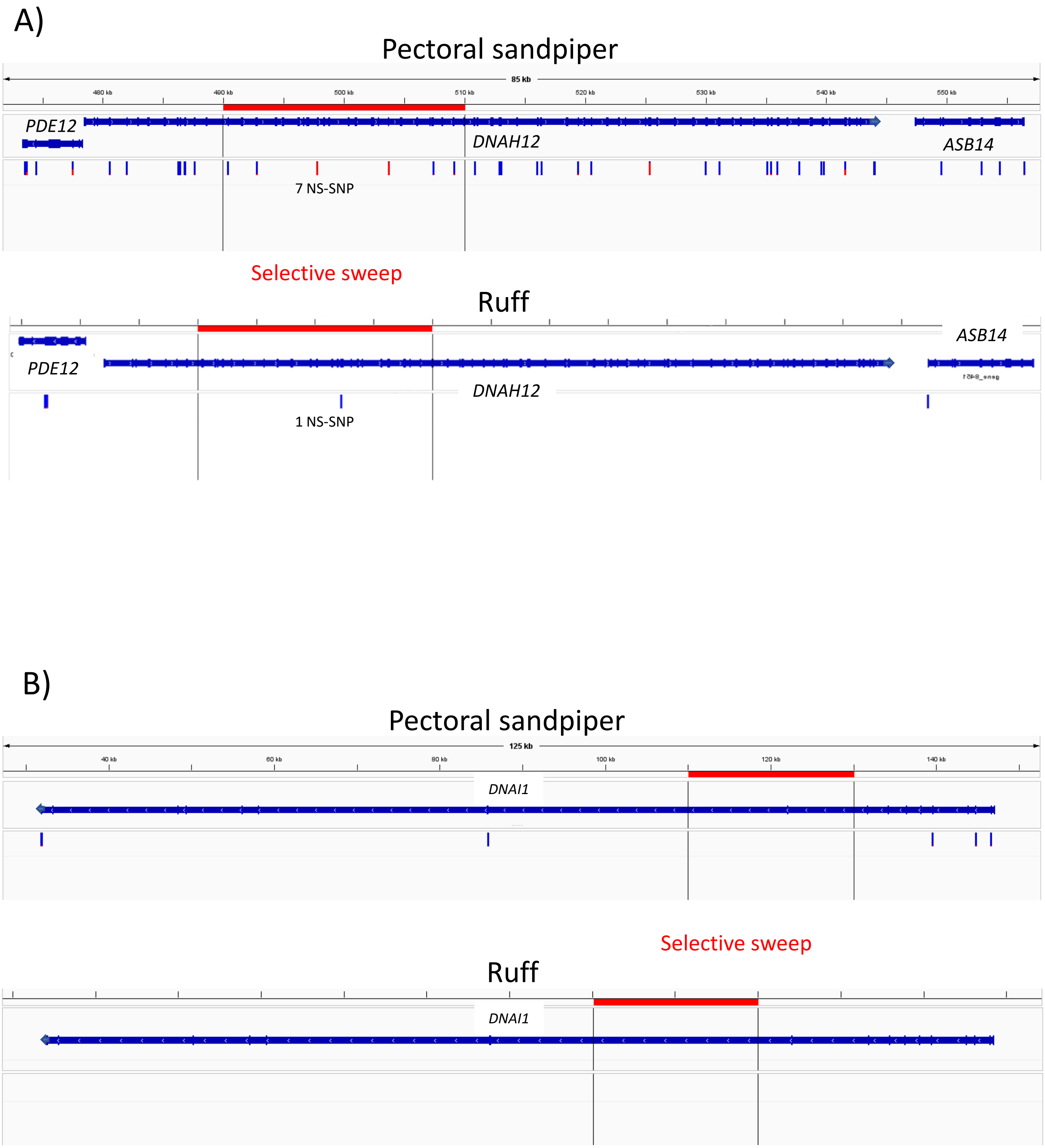

Supplementary figure 4

C)

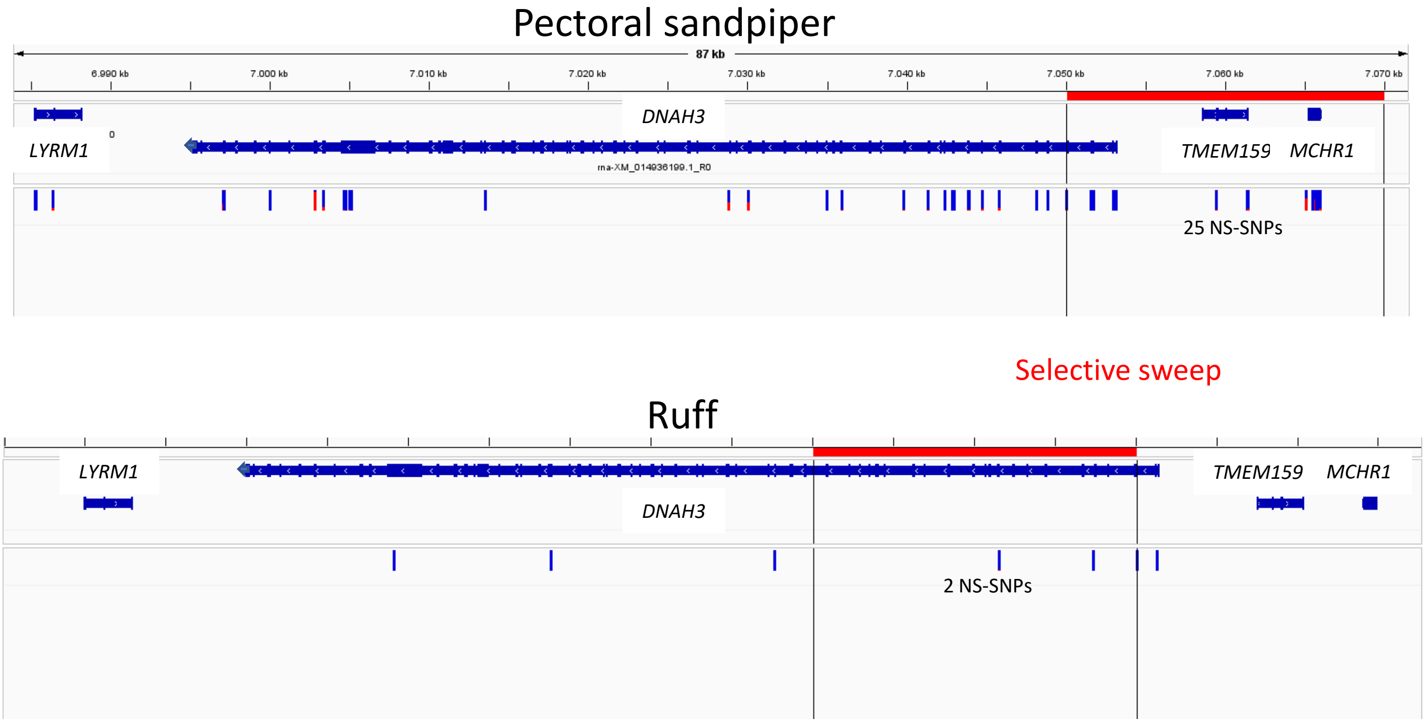

D)

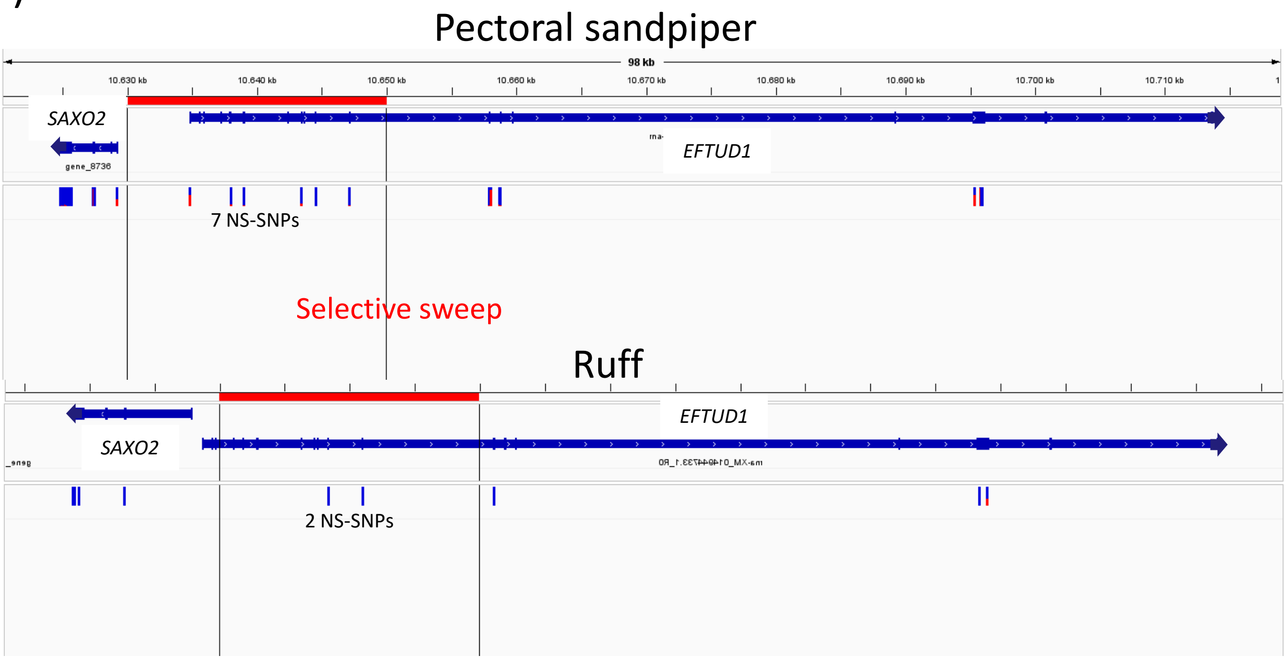

Supplementary figure 4

E)

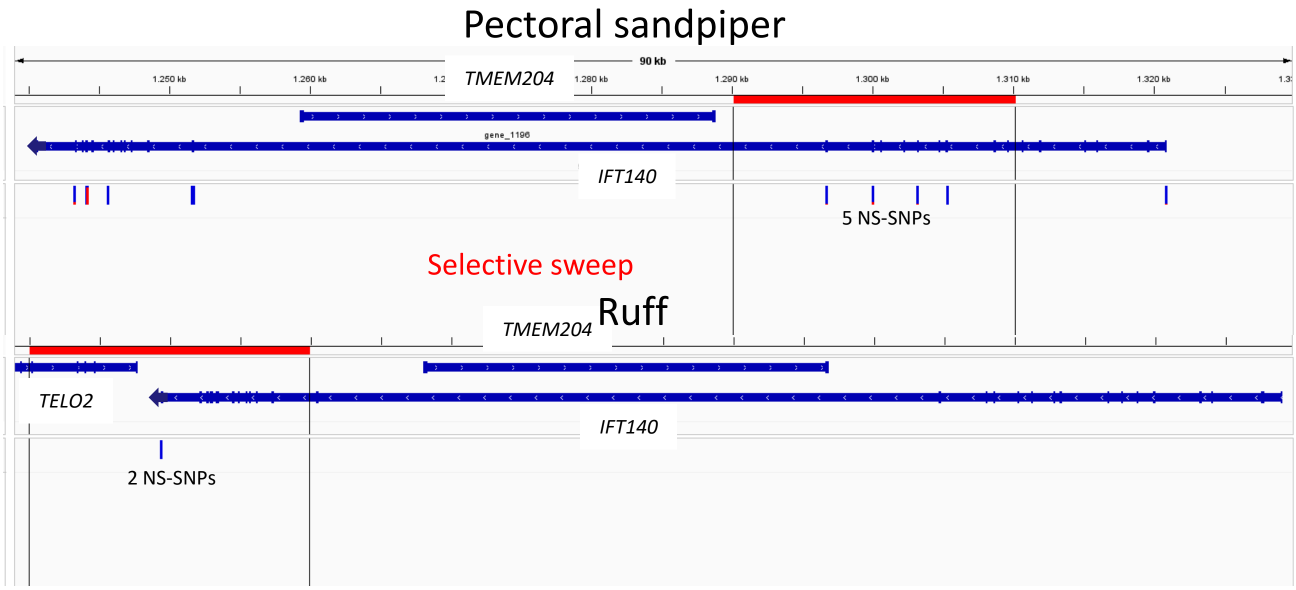

Suppl. figure 4. Genomic regions indicating the selective sweep window and the detected non-synonymous SNPs (NS-SNPs) for the pectoral sandpiper and the ruff in the genes *DNAH12* (A), *DNAI1* (B), *DNAH3* (C), *SAXO2* (D) and *IFT140* (E). Note that closely located NS-SNPs cannot be discriminated in the plot. The colour in each SNP symbol represents the allele frequency (blue = reference allele, red = alternative allele).

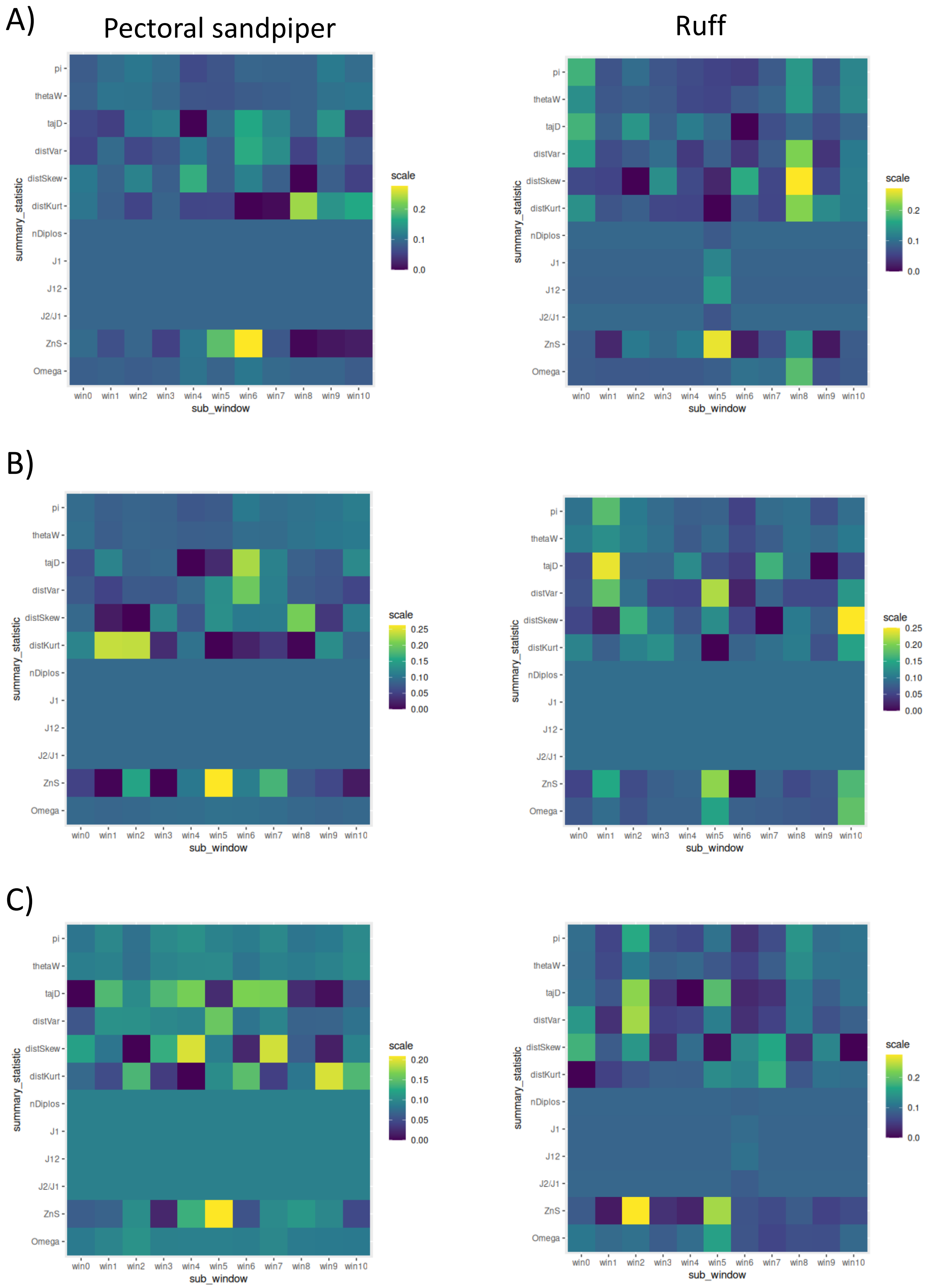

Supplementary figure 5

D)

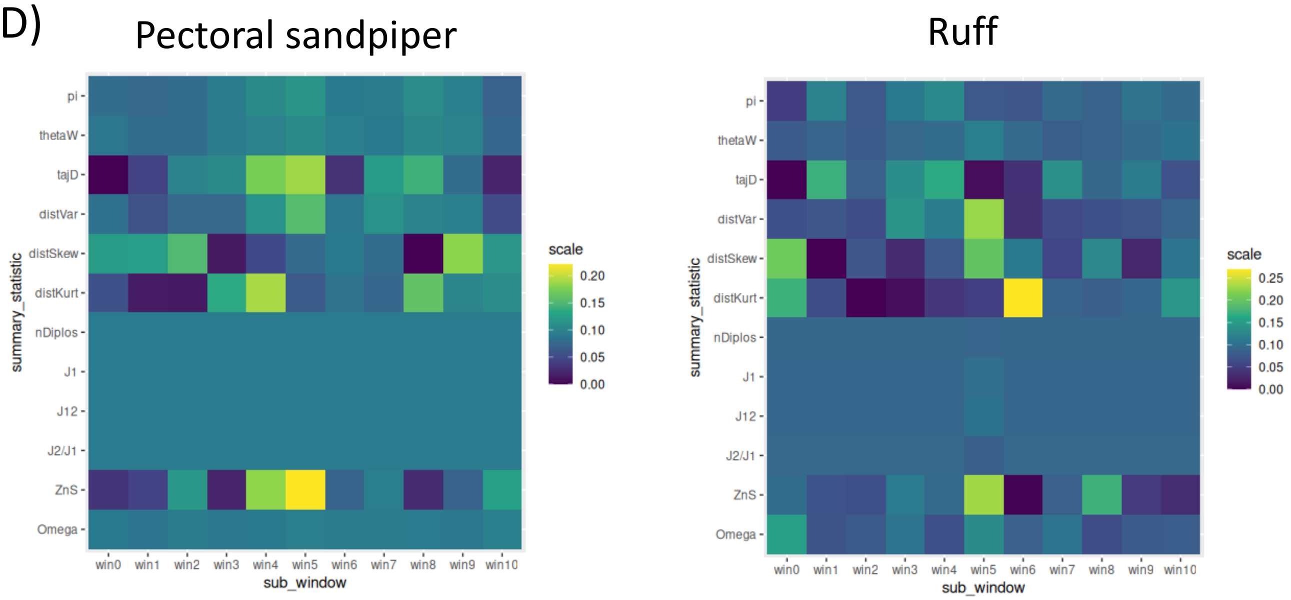

E)

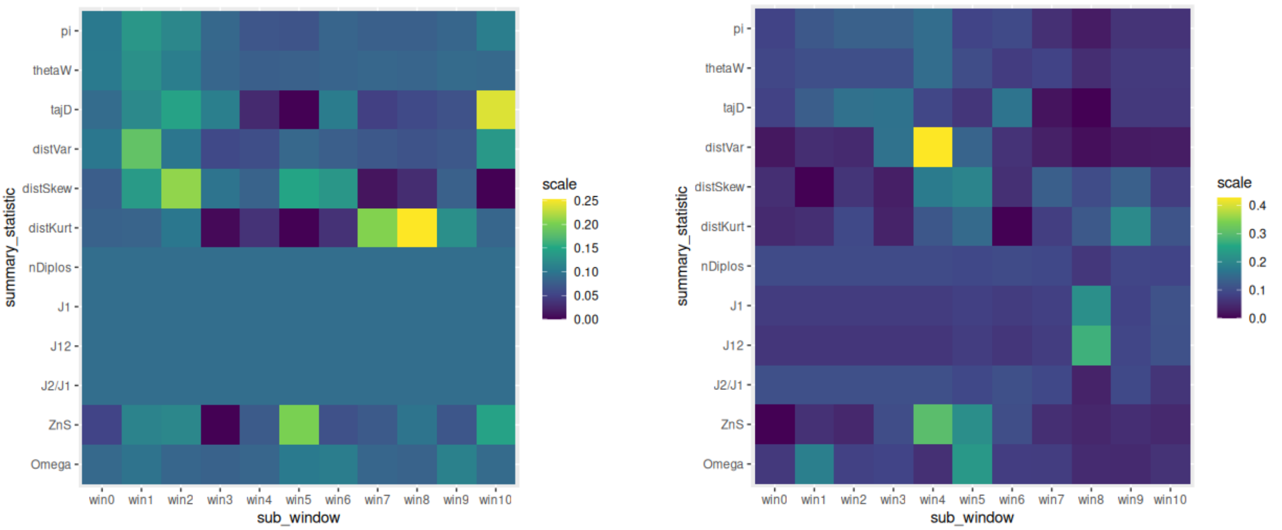

Suppl. figure 5. Heatmaps of the twelve summary statistics (as normalized values) used for the selection scans in the selective sweep window (win5) and ten neighbouring 20-kb windows for the pectoral sandpiper and the ruff in the genes *DNAH12* (A), *DNAI1* (B), *DNAH3* (C), *SAXO2* (D) and *IFT140* (E). The summary statistics are π (pi), θ_w (thetaW), Tajima's D (tajD), genotype distance variance (distVar), genotype distance skew (distSkew), genotype distance kurtosis (distKurt), number of multilocus genotypes (nDiplos), multilocus genotype equivalents of H_1, H_{12} and H_2/H_1 (J_1, J_{12}, J_2/J_1, respectively), unphased Z_{nS} (ZnS), and unphased ω (Omega) (see Kern & Schrider 2018).

Supplementary table 1. Functional enrichment in selective sweep regions of the pectoral sandpiper according to the Wilcoxon rank-sum test of mean sweep probabilities per gene. Panther GO-Slim annotation with parent terms indented. All terms with FDR < 0.05 are listed. Genes with mean sweep probability in upper 20th percentile shared with ruff are in bold.

GO-term	FDR	Annotated genes	Genes with mean sweep prob. in upper 20th percentile
Axoneme (CC)	0.0039	20	*DNAI4, NME7, INPP5E,* ***SAXO2****, TRAF3IP1, HYDIN,* ***DNAH12****,* ***DNAI1****,* ***DNAH3****,* ***IFT140****, DNAH7*
Ciliary plasm (CC)	0.0026	20	*DNAI4, NME7, INPP5E,* ***SAXO2****, TRAF3IP1, HYDIN,* ***DNAH12, DNAI1, DNAH3****,* ***IFT140****, DNAH7*
Plasma membrane bounded cell projection cytoplasm (CC)	0.0083	21	*DNAI4, NME7, INPP5E,* ***SAXO2****, TRAF3IP1, HYDIN,* ***DNAH12, DNAI1, DNAH3****,* ***IFT140,*** *DNAH7*
Cytoplasmic region (CC)	0.0073	31	*DNAI4, NME7, INPP5E,* ***SAXO2****, TRAF3IP1, HYDIN,* ***DNAH12,*** *PHLDB2,* ***DNAI1, DNAH3****,* ***IFT140****, UNC13C, DNAH7*

Supplementary table 2. Functional enrichment in episodic selection across the phylogeny of six *Calidris* spp. according to the Wilcoxon rank-sum test on inversed p-values (1-p) of the unrestricted branch-site dN/dS tests (BUSTED) per gene. Panther GO-Slim annotation with parent terms indented. All terms with FWER < 0.05 are listed.

GO-term	FWER	Annotated genes	Genes with inversed p > 0.95
Axoneme assembly (BP)	0.036	22	*CFAB100, CLUAP1, DNAH5, NISCH, SPAG16*
Microtubule bundle formation (BP)	0.0005	31	*CCSER2, CFAB100, CLUAP1, DNAH5, NISCH, SPAG16*

Supplementary table 3. Expression of the 5 high-lighted shared genes in adult male ruff tissues and humans, and scaffold position in pectoral sandpiper and ruff genome. Values represent DESeq2-normalized read counts (mean ± sd). Note, brain represents an average over 7 brain areas. Ruff data are from Loveland *et al.* 2025 and human tissue expression data from Fagerberg (2014) on 27 different tissues (NCBI gene db). Human sperm proteome according to Greither *et al*. 2023 and Castillo *et al*. 2018.

	**Adrenals (ruff)**	**Blood (ruff)**	**Brain (ruff)**	**Liver (ruff)**	**Pituitary (ruff)**	**Testis (ruff)**	**Tissues with highest expression in humans**	**Gene in human sperm proteome**	**Scaffold in pectoral sandpiper**	**Scaffold in ruff**
**DNAH12**	326 ± 357	63 ± 16	324 ± 199	17 ± 11	701 ± 222	7473 ± 2955	1.testis, 2.lung	Yes	191	JAWSUA010000096.1
**DNAI1**	215 ± 275	12 ± 7	228 ± 114	2 ± 2	108 ± 39	7281± 2117	1.testis, 2.lung	yes	281	JAWSUA010000341.1
**DNAH3**	135 ± 112	2 ± 4	402 ± 284	3 ± 4	451 ± 199	20141 ± 8830	1.testis, 2.lung	yes	271	JAWSUA010000054.1
**SAXO2**	390 ± 528	1 ± 1	152 ± 56	10 ± 3	155 ± 30	1403 ± 361	1.lung, 2.testis	no	80	JAWSUA010000113.1
**IFT140**	1307 ± 244	94 ± 34	1451 ± 347	774 ± 165	3174 ± 422	4222 ± 823	1.testis, 2.thyroid	yes	197	JAWSUA010000048.1
